# The Importance of Nidotherapy and Environmental Change in the Management of People with Complex Mental Disorders

**DOI:** 10.3390/ijerph15050972

**Published:** 2018-05-13

**Authors:** Peter Tyrer

**Affiliations:** Centre for Psychiatry, Imperial College, London W12 0NN, UK; p.tyrer@imperial.ac.uk

**Keywords:** nidotherapy, environmental change, forensic psychiatry, milieu therapy, therapeutic community, cost-effectiveness

## Abstract

Much has been done in the last 50 years to achieve a better understanding of the psychosocial causes and other factors influencing the manifestation of mental illness, but there has been a conspicuous omission. Although gross environmental deficiencies were exposed in old mental institutions, 70 years ago the more subtle maladaptive settings that reinforce chronicity in mental illness have often been forgotten. In this review, the potential of systematic environmental manipulation as a treatment (nidotherapy) and other similar forms of management, used many times in the past but now mainly in forensic settings, is examined. There is now accumulating evidence, reinforced by controlled trials, that planned environmental change, preferably carried out with the full cooperation of the patient, can be a major contributor to therapeutic benefit. It is also very cost-effective. All forms of the environment, physical, social and personal, can be addressed in making assessments, and once a planned way forward has been chosen, progress can be monitored by personnel with limited mental health experience. These interventions have applications in general mental health and occupational health services and deserve much wider use.

## 1. Introduction

The malign influence of negative environments in promoting chronicity in people with mental illness was exemplified most clearly by Erving Goffman [1]. Although the early mental hospitals of the 19th century were forward thinking and innovative, by the middle of the 20th century, the function of many had degenerated into institutions that merely warehoused patients and exercised excessive control. These hospitals were run under an authoritarian system that promoted passivity and inaction, promoted a notion of permanent incapacity, and led people to feel inferior, inadequate, and hopeless. 

The necessary process of deinstitutionalisation followed, initiated by pioneers in services for the intellectually disabled [2], and followed shortly afterwards by the community psychiatry movement. This was a time of great therapeutic enthusiasm and there was a gradual dismantling of the coercive elements of these settings, that forced patients to define themselves as mentally ill, change their thinking and behaviour, suffer humiliations, accept restrictions, and adjust to institutional life. The Goffman book is probably the most widely known and quoted of these various qualitative studies critical of the mental hospital. Indeed, it has come to represent the whole genre as it is the most damning and negative in tone. 

The bleak picture painted by Goffman should be compared with the earlier Victorian views about the use of the mental hospital as a therapeutic setting, in which removal from the polluted squalor of poor urban environments could be replaced by country air, beautiful views of landscapes and parks, a cheerful atmosphere, soft gentle sounds and the warmth of friendship in congenial surroundings. This was an essential part of the treatment programme as there was very else to offer the patients.

However, all this was forgotten as mental hospitals became overcrowded, therapeutic enthusiasm diminished and warehousing became the unfortunate norm [3]. And so mental hospitals moved from places of reform to ones of disposal and became ripe for the criticisms of Goffman and others. But the notion of the environment as an agent of change had not disappeared, and its thread has persisted. 

**Figure 1:**
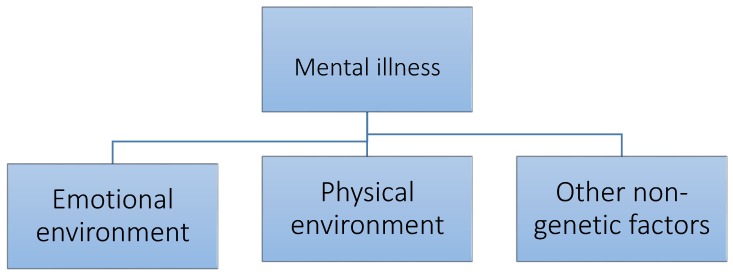
Environmental risk factors responsible for creation of mental illness.

**Figure 2:**
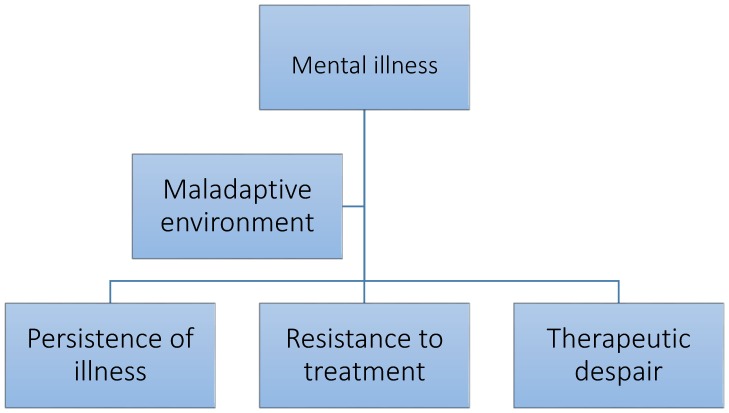
Environmental influence affecting continuation of mental illness.

The focus of care has altered considerably in the past fifty years. There is currently a great deal of research examining environmental risk factors, gene–environmental interactions, and on the importance of life events in generating mental illness. All these are worthy endeavours and have yielded much new information that has aided understanding. Almost all the studies have been focused on the environment as a risk factor for mental illness (Figure 1). The opposite approach examining the use of the environment as a therapeutic factor in mental illness, has attracted less attention (Figure 2). This paper is concerned with exploring the second approach in more detail. 

## 2. Short-Term Intensive Environmental Interventions for Mental Illness 

Under this heading, many forms of environmental intervention carried out in the process of treating patients with a range of mental illness can be described. The adjective “short-term” may seem a little inaccurate but all the treatments concerned are given in specific settings with a view to improving mental health but with no long-term environmental focus in mind, but with the expectation that any relief offered might extend to the longer term.

## 3. Therapeutic Communities and Milieu Therapy 

One of the oldest structured environmental treatments is the therapeutic community, which itself owes a great deal to milieu therapy. Milieu therapy is a form of treatment linked to the principles of therapeutic communities that has gone out of fashion in recent years [4]. It was popular at a time when in-patient stays in mental hospitals were much longer than they are today. It involved giving patients responsibilities, reducing the impact of hierarchies in care, group meetings on wards where staff interacted with patients as equals, and encouragement to make decisions. It was defined by Ellsworth et al [5] as “modification of the environmental part of the patient-environment process with a view to facilitating more satisfactory patterns of interaction”. It did not specify exactly what these changes should be, but its general principles were to involve patients more in decision making, to encourage participation in groups, and to have a flattened hierarchy of control so that doctors’ and nurses’ view could be challenged. It did not claim to effect change beyond the in-patient setting, at least at first. 

Research on the effectiveness of milieu therapy has been generally poor, but this largely reflects the methodology of the time. The best published papers were comparisons of assessment in wards with and without milieu therapy, but these showed no difference in outcomes [6] or better response with behavioural management [7]. Some of the possible adverse consequences of milieu therapy was identified by Vaglum and his colleagues [8]. They concluded that for milieu therapy to be effective individual focused treatment should be preferred to groups, and when group therapy was used it should be more structured with less overt antagonism and criticism. 

Therapeutic communities are linked to milieu therapy and partly developed from them. They are structured, psychologically informed environments that are designed to improve mental health. The notion behind them is a compelling one; that people with a range of emotional and other psychiatric problems, particularly personality ones, can be helped to become more adjusted and prosocial by being placed in an appropriate setting. There was a quasi-political aspect to them also. In the words of Maxwell Jones, one of the popularisers of the movement, “the domination of the doctors was replaced by open communication of content and feeling, information sharing, shared decision making, and problem solving shared as far as possible with all patients and staff” [9]. The essential elements were formulated in an influential study by Rapoport [10] as attachment (sense of belonging), containment (safety), communication (openness), involvement (participation) and agency (empowerment). 

Nobody could disagree with these elements in principle and this explains the persistence of the notion of therapeutic communities as a force for good. But although the theoretical principles of therapeutic communities were established many years ago they have been refashioned many times. One of the reasons for these changes is uncertainty over their key requirements. This is accentuated by the relative lack of organized research into their effectiveness, as although there have been many quasi-experimental studies of their value over the years [11] there has been a long-standing reluctance to carry out randomized controlled studies [12]. These are needed to establish which of the components of the intervention are core ones and which are peripheral and probably unnecessary, and they have been underused, particularly in forensic settings [13]. This has recently been remedied by the publication of the first randomised trial in a day hospital setting. This was a study carried out with 70 patients with personality disorder randomized to normal day hospital care or a day democratic therapeutic community. The primary outcome of days in hospital showed no difference between groups but, of the secondary outcomes assessed at 24 months, satisfaction with care and aggressive behaviour were significantly improved in the therapeutic community group compared with the control population [14].

## 4. Improving Prison Environments

The principles of therapeutic communities became more and more attached to the treatment of personality disorders as solutions to the management of these disorders became more difficult to achieve. This also extended to those with antisocial personality disorders in forensic settings, and one prison, Grendon Underwood in Buckinghamshire, UK, has concentrated on this approach for many years [15]. There is little doubt that the benefit is achieved in this type of setting [16] but as the prisoners are selected for this environment it is possible that they would have a good prognosis anyway.

In recent years the UK has taken this one step further with the development of a new offender personality disorder strategy [17] in which a key component is PIPEs (psychologically informed planned environments (PIPEs) [18]. PIPEs, because they are in custodial settings, are specially designed, contained environments which are not only physically a little different from other prison settings but also have a different social environment. Staff who work there receive additional training to develop better psychological understanding of those under their care. The intention is to create a safe and supportive environment, but not to provide treatment per se. In this respect they can be regarded as a fully-fledged environmental intervention. Evaluation of these is currently being undertaken in this and other settings [19]. 

## 5. Nidotherapy 

Nidotherapy is “the collaborative systematic assessment and modification of the environment to minimise the impact of any form of mental disorder on the individual or on society” [20]. It is named after *nidus* (the Latin word for nest) as the nest is a very good accommodating natural environment. Nidotherapy can be regarded in some ways as an intervention like PIPEs as it is not a treatment. But it differs from PIPEs as the whole focus of management is to change the environment in favour of the person’s wishes, not some external ones. It can be used at any point in the management of a psychiatric patient but is often particularly useful when all other evidence-based interventions have failed [21]. It comprises four phases, personal understanding (an in-depth assessment of the patient’s reasoning and functioning), environmental analysis (of physical, social and personal environments), the development of a timetable of environmental change (the nidopathway), and monitoring and adjustment of the nidopathway. 

## 6. Principles of Nidotherapy

Although the procedure described above may seem a little complicated it need not be, as it is only in the more difficult cases that it is necessary to plot a course through all four stages. In the course of our daily lives we make informed choices that are primarily environmental, such as where we go on holiday, what occupations we choose, and how we choose our partners. This is self-nidotherapy. 

In choosing to practise nidotherapy the practitioner has to be aware of ten principles:

1. All people have the capacity to improve their lives when placed in the right setting.
2. Everyone should have the chance to better themselves.
3. When people become distressed there is always a reason and this is often found in the immediate environment.
4. A person’s environment includes not only place but also other people and self.
5. Seeing the world through another’s eyes gives a better perspective than your eyes alone.
6. What someone else thinks is the best place for a person is not necessarily so.
7. All people, no matter how handicapped, have strengths that should be fostered.
8. There are reasons for all behaviour and many are present in the environment.
9. Every environmental change involves risk.
10. Collaboration is required to change environments for the better.

Special help is needed in nidotherapy when there seems to be no way forward to help patients in terms of their life situation or persistence of unpleasant symptoms or behaviour. This applies most commonly in tertiary psychiatric services when intensive forms of care have been given repeatedly without apparent effect [22]. In choosing what form of environmental change to implement in such cases it is very important to remember the word “collaborative”; changes must always be made with the full approval of the person concerned. 

This involves getting a full understanding of the person’s needs as well as wants, and explains the importance of the first phase of nidotherapy, personal understanding, which is probably the essence of the therapeutic relationship [23]. Once this and the environmental analysis have been completed the changes are planned. Sometimes only a single environmental change may be necessary; more often it is two or three, but they usually link together well. 

Nidotherapy is a form of reverse Darwinism. Instead of waiting for the person to change so it fits the environment, nidotherapy changes the environment so that it fits the person. Darwin originally described natural selection as “survival of the adapted” and if this was to be rephrased to explain nidotherapy it could be described as “*better environmental adaptation in however slight a degree to the mental state conditions*”. So, when the persistently mentally ill fail in normal competition for one environment, they succeed if they find one that suits their particular needs only. 

In this context it is clear that nidotherapy is not just a discipline for the mental health specialist. It is part of public mental health and can be practised by many. Thus, for example, in occupational medicine when there are great difficulties in finding a niche for an unusual employee who has potential benefit, nidotherapy can help to find this position and help both employer and employee [24,25]. 

## 7. Overlap between Nidotherapy and Other Interventions

Nidotherapy differs in several ways from the environmental interventions already described, but it overlaps a little with others. It shares a considerable component with the recovery model [26] in that it is highly patient-orientated and collaborative, but it differs in only focusing on the environment and not attempting to treat illness. It also shares some features with person-centred care in intellectual disability [27], but again it differs in only being focused on environmental change. 

So we have clear demarcation zones for nidotherapy. First, it is not intended to treat mental illness in any of its forms. This may seem paradoxical but by concentrating on environmental change only it is possible to avoid the many issues of mental health interactions that could otherwise create conflict. Clearly mental health is the elephant in the nidotherapy room but by approaching it indirectly more success is achieved [28]. Second, nidotherapy is a long-term environmental intervention. Its time scale is variable, but can be carried out over many years, as it attempts to create a permanent change in a person’s circumstances. Third, it is genuinely collaborative. I use the adverb “genuinely” quite deliberately. Many collaborative endeavours in mental health are not really using collaborative in what I regard as a strictly accurate sense, as they are geared towards the dominance of the health professional in determining the outcome of the collaboration. In nidotherapy the decisions about environmental change are made by the patient. They are made in the understanding that they are owned and maintained by the patient, not just in the present but in the future too. The aim of the nidotherapist is to enhance the resolve and ability of the patient to achieve the environmental changes they wish for and are feasible.

## 8. Evidence Level of Environmental Interventions

It is unfortunate for science that most of the environmental interventions described above have not been tested beyond anecdote and simple description. This is partly because environmental change appears to consist of common sense and serendipity; it has no scientific underpinnings. But this is a mistaken impression. The study of gene–environment interactions, mainly using large existing data-bases, has shown that the measurement of environment is fraught with error and multiple confounders, and whilst the specific generic component can be identified clearly, the environmental ones can only be guessed at or joined together in complex algorithms that have no external validity [29,30,31].

It is not fully appreciated that evaluation of environmental change is a difficult task as it is a very complex intervention [32]. Thus, we only have one published randomised trial of democratic therapeutic communities [14] despite its delineation as a treatment over 70 years ago, none of milieu therapy, none of person-centred therapy, none yet of PIPEs, and although several attempts have been made to test formally the recovery model and day hospital initiatives they have all been inconclusive because of methodological problems [33,34]. 

Nidotherapy has fared a little better. A detailed qualitative study showed it was very difficult to implement in forensic settings [35] (where PIPEs would probably be more appropriate), and there have been two randomised trials, one in an assertive outreach team in those with severe mental illness [36] and the other, a cluster-randomised trial in care homes for adults with intellectual disability and challenging behaviour [37]. Both these trials showed benefit of nidotherapy compared with other active treatment approaches, and also in terms of social function and cost [38]. But they were relatively small trials and as they were preliminary they need replication. There is now sufficient background knowledge to make definitive larger studies both practicable and needed. 

## 9. Taxonomy of Environments and Their Interpretation

In developing treatments focused on the environment a good taxonomy of environmental change is needed. This currently does not exist, and the following only represent a suggested way forward (Table 1). Firstly, there are consensual general environmental changes (CGECs). These are the decisions we make repeatedly in our lives through our own choices and can be summarized as self-nidotherapy (if a label is needed). There are others which apply to large groups or populations and are not related to specific psychiatric problems. Thus, for example, the ’broken windows’ theory of crime that posited more crime and vandalism when windows that had been broken were not replaced immediately [39], is a general environmental change that is said to help or hinder whole communities depending on the response. Similarly, advances in architecture of mental hospitals to adjust to the needs of patients [40,41] can be put into this category, as can innovative activities for groups of people such as Green Care promoted by the Royal College of Psychiatrists [42]. These often offer flexibility in performance if tasks, including suggestions and changes made by people who use them, but they are not primarily initiated by the patients themselves. 

There are other clearly forced environmental changes (FGECs) such as moving prisoners to and from correctional institutions, residents into and from care homes for the frail and disabled, and psychiatric patients into hospital under coercion or away from hospital when there are bed shortages. 

Secondly, there are focused environmental changes (FECs) that are specific to the individual and immediate family or friends. These can be very simple and straightforward, such as moving house, changing jobs or moving in with a partner. These do not need special help beyond discussion with others. The more difficult changes are those that appear to be clearly needed but cannot be attained for a variety of reasons. Here the changes can also be separated into forced focused environmental changes (FFECs) where some attention is paid to the specific needs of the individual concerned but involve little input from the individual themselves, and consensual focused environmental changes (CFECs) where the changes are made with varying degrees of approval from the people concerned. Nidotherapy is specifically concerned with CFECs when there is doubt if the suggested changes made are fully owned and embraced by the person concerned.

The most difficult environmental changes are desired but resistant ones (DRECs) in which either the subject or the controller of the environment cannot agree on their implementation. This may be of doubt or lack of courage by the subject, concern by others over the wisdom of the change (often with risk as a factor in the background), or perceived impracticality of the change. This is where the skills of the nidotherapist are needed most clearly.

Nidotherapy is primarily concerned with CFECs and DRECs. When faced with a set of complex, apparently insurmountable, environmental disadvantages that have been present for a long time it is not easy to find a collaborative way forward. The nidotherapist has to be a guide but not necessarily a leader in this task. Exploring the options has to be both careful and gentle, often needing a considerable amount of tact and diplomacy in dealing with obstacles and yet retaining the trust of the person concerned. The role of environmental advocacy can be an important one in these circumstances.

The resistance to change in DRECs may come from several sources. The patient may be reluctant to change because of lack of courage or fear of failure—this is called the Prufrock syndrome [17] after the indecisive centrepiece of TS Eliot’s poem [43]—or the environmental system may refuse to countenance the desired change. Here the environmental analysis may be relatively straightforward but the execution of the change much more difficult. Currently we have a patient who wishes to break off from a highly controlling relationship, but his fears of abandonment are holding him back. We have needed three meetings, including the latest with his parents and with another patient who has made a similar break after a very long period [28], and now the change has been made. 

It is also important to recognize that the categories of environmental change can also change. One of the main achievements of nidotherapy has been to change forced changes (FFECs) into consensual ones (CFECs) when patients are discharged from psychiatric hospitals. When the correct match is achieved a discharge is not followed by readmission [36] and the service user’s needs are satisfied [44]. 

In evaluating the effectiveness of nidotherapy we have used a fidelity scale [21] but this has limited value in solving the more difficult problems. It is more difficult to evaluate consensual changes than forced ones as, in nidotherapy, the exact parameters of intervention are determined by the patient, not by the specific nature of the intervention. For this reason, it is not easy to set up trial designs to test efficacy; cluster randomized trials may have an important place here [37,45].

## 10. Conclusions

Environmental interventions in mental illness have been known for more than a century but are only now being assessed as serious subjects for careful evaluation. In this review, nidotherapy an intervention designed to change the environment only, is compared with others that overlap with it to varying extents. Their relative merits still need better controlled comparisons with a better taxonomy of environmental factors. 

## Figures and Tables

**Table 1:** Taxonomy of environmental changes and their implementation.

Type of Environmental Change	Tasks Needed to Complete	Ease of Completion
Consensual general environmental changes (CGECs)	Individual decision-making with friends and family if needed	Easy
Forced general environmental changes (FGECs)	External organisations determine change unilaterally (e.g., move from prison)	Easy
Forced focused environmental changes (FFECs)	Decisions made by external agencies with little or no input from individual	Relatively easy, especially in coercive situations
Consensual focused environmental changes (CFECs)	Agreed changes made in full cooperation and with agreement of individual	Fairly easy to fairly difficult, depending on nature of change *
Desired but resistant environmental changes (DRECs)	Environmental advocacy and persuasion of all parties to agree to changes	Very difficult *

* Involvement of nidotherapy.
